# Metabolomic and proteomic differences in susceptible and benzimidazole-resistant adult females and males of *Haemonchus contortus*

**DOI:** 10.1186/s13567-025-01698-3

**Published:** 2025-12-24

**Authors:** Helena Pelantová, Michaela Šadibolová, Martin Žofka, Petra Matoušková, Marcin Luzarowski, Josef Krátký, Karolína Štěrbová, Marek Kuzma, Ondřej Vosála, Lenka Skálová

**Affiliations:** 1 Laboratory of Molecular Structure Characterization, Institute of Microbiology, Czech Academy of Sciences, Vídeňská 1083, Prague, Czech Republic; 2 Biomedical Research Centre, University Hospital Hradec Králové, Hradec Králové, Czech Republic; 3 Department of Biochemical Sciences, Faculty of Pharmacy, Charles University, Heyrovského 1203, Hradec Králové, Czech Republic; 4 Core Facility for Mass Spectrometry and Proteomics, Center for Molecular Biology of Heidelberg University (ZMBH), Heidelberg, Germany

**Keywords:** Parasitic nematodes, metabolome, proteome, anthelmintic resistance, resistance mechanisms, sex differences

## Abstract

Anthelmintic resistance in parasitic nematodes, particularly in *Haemonchus contortus*, poses a significant threat to livestock health and productivity. Since resistance mechanisms have not yet been fully elucidated, the present study employed metabolomic and proteomic analyses of *H. contortus* adults (females and males separately) from drug-susceptible (ISE) and benzimidazole-resistant (IRE) strains, with a focus on resistance-specific differences. Using nuclear magnetic resonance (NMR)-based metabolomics and mass spectrometry-based proteomics, significant alterations in metabolic and protein expression profiles associated with resistance were identified. Resistant adults exhibited reduced levels of multiple amino acids and tricarboxylic acid cycle intermediates, alongside elevated levels of trehalose, myo-inositol, NAD^+^, and glycerophosphocholine, suggesting enhanced stress resistance and altered energy metabolism. Proteomic analysis revealed over 3000 proteins, with substantial sex-related differences. Moderate resistance-related differences in protein expression were observed, indicating adaptive biochemical pathways supporting resistance. While some resistance-associated changes in the metabolome and proteome were observed in both sexes, several were distinctly sex-specific. This finding provides the first evidence of such sex-dependent strategies in *H. contortus*, reinforcing the notion that anthelmintic resistance is a multifaceted and highly complex phenomenon, with many aspects yet to be fully understood. In addition, certain nematode-specific enzymes upregulated in the IRE strain - such as cysteine synthases and transthyretin-like proteins - might be considered as potential targets for future anthelmintic development.

**Supplementary Information:**

The online version contains supplementary material available at 10.1186/s13567-025-01698-3.

## Introduction

Parasitic nematode infections exert a substantial global burden, adversely affecting the health of billions of individuals and compromising livestock productivity [1]. In response to this challenge, extensive research has been conducted into the physiology and biochemistry of nematodes, aiming to facilitate the development of effective control strategies. Although the free-living *Caenorhabditis elegans* represents the most studied model nematode [2], huge differences in the nematodes with parasitic lifestyle are to be expected. Among parasitic nematodes, *Haemonchus contortus*, an intestinal pathogen of artiodactyls, is by far the most researched model [3, 4], probably owing to its direct and short life cycle (without intermediate host) and relatively easy availability and cultivation. Moreover, *H. contortus*, also known as the barber’s pole worm, represents one of the most common and pathogenic parasite of small ruminants, causing large economic losses for farmers worldwide [5, 6].

Infection with *H. contortus* (called haemonchosis) manifests with anemia, edema, slow growth, weight loss, and, in cases of strong infection, even death of infected animals. Although grazing management, nutrition supplementation, selective breeding, or vaccination are partly helpful in haemonchosis prevention [7–9], chemotherapy is still the mainstay for the treatment of haemonchosis as well as other helminthiases. However, a limited number of anthelmintics are available on the market and the effectiveness of the available anthelmintics has decreased owing to anthelmintic resistance selection in nematodes [10–12]. Therefore, the discovery of novel targets to design new anthelmintics, which will also be effective in resistant strains, are extremely needed. In addition, a comprehensive understanding of the resistance mechanisms is necessary for the characterization of new drug resistance markers, which could be used in anthelmintic resistance diagnostics in field and laboratory populations [13]. Concerning anthelmintic resistance mechanisms, *H. contortus* has attracted particular attention, as this parasite has been able to develop drug resistance in a very short time to structurally diverse anthelmintics [5, 14, 15].

Mechanisms of drug resistance are broadly categorized into target-site and nontarget-site pathways [3, 16]. Target-site resistance in nematodes is particularly well-characterized in the context of benzimidazole anthelmintics, owing to multiple reported mutations in their primary molecular target, β-tubulin isotype I. In *H. contortus*, a single nucleotide polymorphism (SNP) involving a codon change from TTC to TAC at position 200 has been documented, resulting in a phenylalanine-to-tyrosine substitution [17]. This mutation has been associated with a diminished binding affinity of benzimidazoles to β-tubulin. Furthermore, additional SNPs at codons 167 and 198 have also been implicated in the development of benzimidazole resistance [18–20]. Interestingly, population-wide allele sequencing revealed the presence of distinct β-tubulin alleles that remained stable within the population even after the withdrawal of benzimidazole selection pressure [21, 22]. Although mutations in the benzimidazole target β-tubulin represent the primary driver of benzimidazole resistance, new alleles for resistance to benzimidazoles have been identified recently [23]. Further, nontarget site mechanisms, mainly based on improved deactivation and/or efflux of anthelmintics, can also be involved in benzimidazole resistance in nematodes [24]. In addition, the results of recent “omics” studies [25–27] indicate the existence of resistance-related changes in endogenous biochemical pathways.

In the present study, metabolomic and proteomic analysis was combined to gain a deeper understanding of this issue. The metabolome and proteome of *H. contortus* adults from benzimidazole-susceptible and benzimidazole-resistant strains were compared to reveal resistance-related metabolites and enzymes that could help the parasite to survive under stress evoked by the contact with the drug. An inbred susceptible-Edinburgh strain (ISE; MHco3) and an inbred resistant-Edinburgh strain (IRE; MHco5), derived from the ISE strain by benzimidazole selection pressure [28], were used. Adult females and males of *H. contortus* were analyzed separately to address sex-specific resistance-related differences in protein expression and metabolite levels.

## Materials and methods

### Sheep breeding and infection

Prior to our study, lambs were dewormed with a single dose of albendazole (*per os* 5 mg/kg). The effectiveness of deworming was checked using coprological analysis. Six parasite-free male sheep (Texel breed, 6 months old) were orally infected with 8000 L3 larvae (suspended in 5 mL of water) of *H. contortus*. Two strains of *H. contortus* were used: the ISE strain (MHco3) and the IRE strain (MHco5), which were purchased from Moredun Research Institute (UK). The IRE strain was prepared from the ISE strain by benzimidazole selection pressure [28, 29]. The animal protocols were conducted in accordance with the Guide for the Care and Use of Laboratory Animals (Protection of Animals from Cruelty Act no. 246/92, Czech Republic). The breeding facilities were accredited by the Ministry of Agriculture of the Czech Republic for experimental sheep housing (MZE-53255/2022–13143). All experimental procedures involving sheep were evaluated and approved by the Ethics Committee of the Ministry of Education, Youth and Sports of the Czech Republic (project number MSMT-20144/2023–4).

### Isolation of *H. contortus* adults

Seven weeks after infection, the lambs were stunned and exsanguinated in agreement with Czech slaughtering rules for farm animals The isolation of adults was conducted using the agar method as previously described [30]. The sex of *H. contortus* adults was determined via microscopic examination of morphological features. The separation of males and females was done manually. The samples for metabolomic and proteomic analyses were prepared immediately after separating males and females.

### Samples preparation for metabolomic analysis

For this study, four experimental groups were established, each comprising ten replicates: ISE males, ISE females, IRE males, and IRE females. Each sample was prepared from 10 females or 20 males. The worms were quick-frozen in liquid nitrogen and powdered using a micro-homogenizer (Bel-Art^®^ Disposable Pestles, Merck, Czech Republic) in a centrifuge tube. Then, 1 mL ice-cold methanol was added directly to the powdered tissue and vigorously shaken for 5 min, next the extract was centrifuged (10 min, 16 000 × *g*). The supernatant was transferred to microtubes, evaporated in a rotary vacuum concentrator (Eppendorf Concentrator Plus, Sigma-Aldrich, Czech Republic), and stored at −80 °C until nuclear magnetic resonance (NMR) analysis.

### NMR-based metabolomic analysis

Dry extracts were thawed at room temperature, mixed with 180 µL D_2_O and 20 µL phosphate buffer (1.5 M KH_2_PO_4_ in D_2_O containing 2 mM NaN_3_ and 0.1% trimethylsilyl propionic acid [TSP], pH 7.4), and transferred to 3-mm NMR tubes. The NMR spectra were acquired on a 600 MHz Bruker Avance III spectrometer (Bruker BioSpin, Rheinstetten, Germany) equipped with a 5 mm TCI cryogenic probe head. All experiments were performed using Topspin 3.5 software at 25 °C with automatic tuning and matching, shimming, and optimization of the 90° pulse length for each sample. The proton spectra were recorded using the Carr–Purcell–Meiboom–Gill (CPMG) pulse sequence (cpmgpr1d), incorporating presaturation during the relaxation delay d1 (4 s), with the following parameters: number of scans (NS) = 512, number of data points (TD) = 64 k, spectral width (SW) = 20 ppm, echo time = 0.3 ms, and loop for T2 filter = 1260. The CPMG sequence was selected to suppress broad resonances and improve spectral baselines. Metabolite assignments were supported by additional 2D NMR experiments (J-resolved, HSQC, and TOCSY) conducted on representative samples. Raw spectral data were processed in TopSpin 3.5 software (Bruker BioSpin, Rheinstetten, Germany). Free induction decays (FIDs) were multiplied by an exponential window function (LB = 0.3 Hz) before Fourier transformation, and were automatically phased and referenced to the TSP signal. Spectral regions between 0.7 and 10.0 ppm (excluding the residual water peak) were normalized to the total spectral area using the open-source software NMRProcFlow 1.4 [31].

### NMR data evaluation

For untargeted analysis, normalized spectra were segmented into equidistant bins of 0.04 ppm width. Multivariate analyses were performed using Metaboanalyst 6.0 software [32]. Principal component analysis (PCA) was conducted on mean-centered and Pareto-scaled data to examine sample clustering and detect outliers. Partial least squares-discriminant analysis (PLS-DA) was then employed to build classification models, which were validated using leave-one-out cross-validation (LOOCV) and permutation testing. The results of the PLS-DA model were evaluated using variable importance in projection (VIP) scores, which identified bins contributing most significantly to group separation. All well-resolved non-overlapping signals or multiplet parts were subjected to targeted metabolomic profiling. Metabolite identification was carried out using Chenomx NMR Suite (Chenomx Inc., Edmonton, Canada), supplemented by literature data and confirmed by comparison with chemical shift information from the Human Metabolome Database (HMDB) and the Biological Magnetic Resonance Data Bank (BMRB). Structural assignments were further supported by J-resolved and TOCSY spectra and validated using carbon chemical shifts from HSQC spectra. Univariate statistical significance was assessed using a two-way analysis of variance (ANOVA), followed by Tukey’s post hoc test for pairwise comparisons, with the Benjamini–Hochberg procedure applied to control the false discovery rate (FDR). Differences in metabolite concentrations with *p*-values below 0.05 were considered statistically significant.

### Protein extraction

Proteins were extracted from adults of *H. contortus* in four biological replicates. Briefly, 10 female and 20 male adults were separately transferred to clean tubes with 600 µL of lysis buffer (8 M urea in 100 mM tetraethylammonium bromide (TEAB), pH 8.5) and homogenized using a FastPrep-24 5G Homogenizer (MP Biomedicals, Illkirch-Graffenstaden, France) during two cycles of 40 s at 6 m/s. Homogenized samples were transferred to new tubes and further disintegrated with three 30-s cycles of sonication (Sonoplus HD 2070, Bandelin, Berlin, Germany). Samples were centrifuged at 12 000 × *g* for 20 min at 4 °C. Protein concentration of supernatants was estimated using Bradford assay (Bio-Rad, Hercules, CA, USA) according to the manufacturer’s instructions.

### Protein digestion

In total, 600 µg of protein was precipitated using chloroform–methanol precipitation [33]. Protein pellets were resuspended in 40 µL of 8 M urea (in 100 mM TEAB), and protein concentration was estimated using the Bradford assay. Afterward, 150 µg of protein was reduced and alkylated with 10 mM tris(2-carboxyethyl)phosphine hydrochloride (TCEP) and 40 mM 2-chloroacetamide (CAA), respectively, for 30 min at room temperature. Samples were first digested with lysyl endopeptidase (1:50 *w*/*w*; FUJIFILM Wako Pure Chemical Corporation, Osaka, Japan) for 4 h at 37 °C and then diluted with 50 mM TEAB (final urea concentration of 1.6 M) and digested with trypsin (1:50 *w*/*w*; Thermo Fisher Scientific, Waltham, MA, USA) overnight at 37 °C. After digestion, samples were acidified with trifluoracetic acid (TFA) to a final concentration of 0.8% (*v*/*v*). Samples were desalted using Sep-Pak tC18 100 mg cartridges (Waters, Milford, MA, USA). Peptides were eluted with 50% acetonitrile (ACN)/0.1% TFA followed by 80% ACN/0.1% TFA, and vacuum-dried.

### TMT labeling

Peptides were resuspended in 30 µL of 100 mM TEAB. For intensity normalization, a pooled sample was generated by mixing equal aliquots of all digested samples prior to TMT labeling. TMT reagents (0.8 mg; Thermo Fisher Scientific, Waltham, MA, USA, LOT: UL289970) were dissolved in 70 µL of ACN. For labeling, 20 µL of samples were mixed with 8.2 µL of TMT reagents (final TMT concentration of 3.3 µg/µL) and incubated for 1 h at 25 °C. Labeling was quenched with 2.5 µL of 5% hydroxylamine for 15 min at 25 °C. Labeling efficiency and normalization factors within created multiplexes were checked by 70-min and 105-min DDA runs, respectively. Final multiplexes were acidified with TFA to a final concentration of 0.8% (*v*/*v*) and desalted as described above. The TMT experiment quality check is shown in Additional file 1, and TMT experimental design is provided in Additional file 4.

### High pH reversed-phase fractionation

TMT-labeled multiplexed samples were dissolved in 10 mM ammonium formate (pH 10) and fractionated using an Acclaim PepMap 100 C18 HPLC column (2 µm, 300 µm × 150 mm; Thermo Fisher Scientific, Waltham, MA, USA) on a Vanquish Neo UHPLC system (Thermo Fisher Scientific, Waltham, MA, USA) controlled by Chromeleon Chromatography Data System [34, 35]. The instrument was operated at a constant flow of 3 µL/min with eluent A (10 mM ammonium formate, pH 10) and eluent B (10 mM ammonium formate in 90% ACN, pH 10). Peptides were loaded onto the column using a FlowControl mode (with enabled Fast Loading) at 3% B and separated using the following gradient: 0–5 min, 3% B; 5–17 min, 3% B to 40% B; and 17–20 min, 40% B to 70% B. The column was washed by two consecutive washes with 95% B and equilibrated at 3% B using a FlowControl mode (with enabled Fast Equilibration) for 7 min. Forty fractions were collected from 7 to 27 min and concatenated into eight final fractions (30-s collection, five cycles) [36]. The quality of high pH fractionation was monitored using absorbance at 214 nm. Fractions were subsequently dried using a centrifugal evaporator.

### LC–MS/MS proteomics analysis

Acetonitrile (product no. 012041 01), formic acid (product no. 069141 43), and water (product no. 232141 B1) used for the liquid chromatography-tandem mass spectrometry (LC–MS/MS)-related procedures were of the highest quality and were acquired from Biosolve (Valkenswaard, the Netherlands). Trifluoroacetic acid was acquired from Merck (TFA; 302031, Darmstadt, Germany). LC–MS/MS analysis was performed on a Dionex UltiMate 3000 RSLCnano HPLC system coupled with a QExactive HF orbitrap mass spectrometer (both from Thermo Fisher Scientific, Bremen, Germany). Peptide separation was achieved on an in-house packed C18 column (75 µm × 230 mm, ReproSil-Pur 120 C18-AQ, 1.9 µm particle size; Dr Maisch, Ammerbuch-Entringen, Germany). Eluent A consisted of 0.1% formic acid in 1% ACN (*v*/*v*) and eluent B consisted of 0.1% formic acid in 89.8% ACN (*v*/*v*). Fractionated samples were dissolved in 0.1% TFA and loaded onto a column for 20 min at a flow rate of 0.55 µL/min and 3% B. Peptides were eluted during a two-step linear gradient increasing from 3% B to 23% B in 21 min and further to 38% B over 8 min at a reduced flow rate of 0.3 µL/min, followed by two consecutive washes with 95% B and reconditioning to 3% B. The nanospray ionization source was operated using the following settings: spray voltage 2.5 kV, capillary temperature 250 °C, probe heater temperature 350 °C, and S-lens RF level 60. Full-scan MS spectra were recorded in a profile mode within a scan range of 400–1600 *m*/*z*, with a resolution of 60 000, AGC target of 3e6, and maximum injection time of 50 ms. The top 15 precursors were isolated using the isolation window of 0.7 *m*/*z* and fragmented with the normalized collision energy of 32%. MS2 spectra were acquired at a resolution of 30,000, ACG target of 1e5, and maximum injection time of 100 ms. Dynamic exclusion was set to 10 s.

### Proteomic data processing

Raw files were searched in Proteome Discoverer (v3.0). For each TMT multiplex, raw files from the eight fractions were merged and searched with the Sequest HT [37] search engine against the *H. contortus* protein database (UniProt UP000025227; 20,964 entries) and *Ovis aries* (sheep) protein database (UniProt UP000002356; 21,221 entries) both downloaded in February 2025. Briefly, Trypsin/P was used as a cleavage enzyme with up to two missed cleavages allowed. Carbamidomethylation (C) and TMT6plex (K) were set as fixed modifications, while oxidation (M), acetylation (protein N-terminus), and TMT6plex (peptide N-terminus) were set as variable modifications. The mass error tolerance for full-scan MS spectra was set to 10 ppm and for MS/MS spectra to 0.1 Da. A target–decoy strategy to control for peptide false discovery and identification was validated by Percolator [38] software. Protein and PSM false discovery rates (FDR) were kept at 1%. Only unique peptides assigned to each protein group were considered for quantification. TMT reporter intensities were adjusted using the corresponding isotopic distribution correction factors provided by the manufacturer.

### Proteomic data analysis

Protein search results were processed in R programming language (v4.4.0 with RStudio v2024.04.0 + 735). Only high-confidence proteins found uniquely in the *H. contortus* protein database were used for further data analysis. Data normalization was performed according to Plubell et al. 2017 [39]. Briefly, the total reporter ion intensity in each channel was first adjusted to the mean total intensity of all channels across all four multiplexes. Subsequently, scaling factors for each individual multiplex were obtained by adjusting the reporter ion intensity of the pooled sample in each individual multiplex to its geometric mean in all four multiplexes. The scaling factors were calculated separately for each protein in each multiplex. Differential abundance analysis was performed using the limma (v3.62.2) R package [40], which applies linear modeling with empirical Bayes moderation, with defined contrasts and a Benjamini–Hochberg FDR of 5%. All proteins that had at least two valid values were used for the statistical analysis. Plots were generated using ggplot2 (v3.5.2) and pheatmap (v1.0.12). Functional over-representation analysis (ORA) was performed using the enricher function from clusterProfiler (v4.14.6) with Benjamini–Hochberg correction (5%). *H. contortus* protein annotation data (Gene Ontology annotation) were downloaded from UniProt in July 2025. All quantified *H. contortus* proteins were used as a background database to calculate the enrichment scores.

## Results

### Metabolome analysis

**Figure 1:**
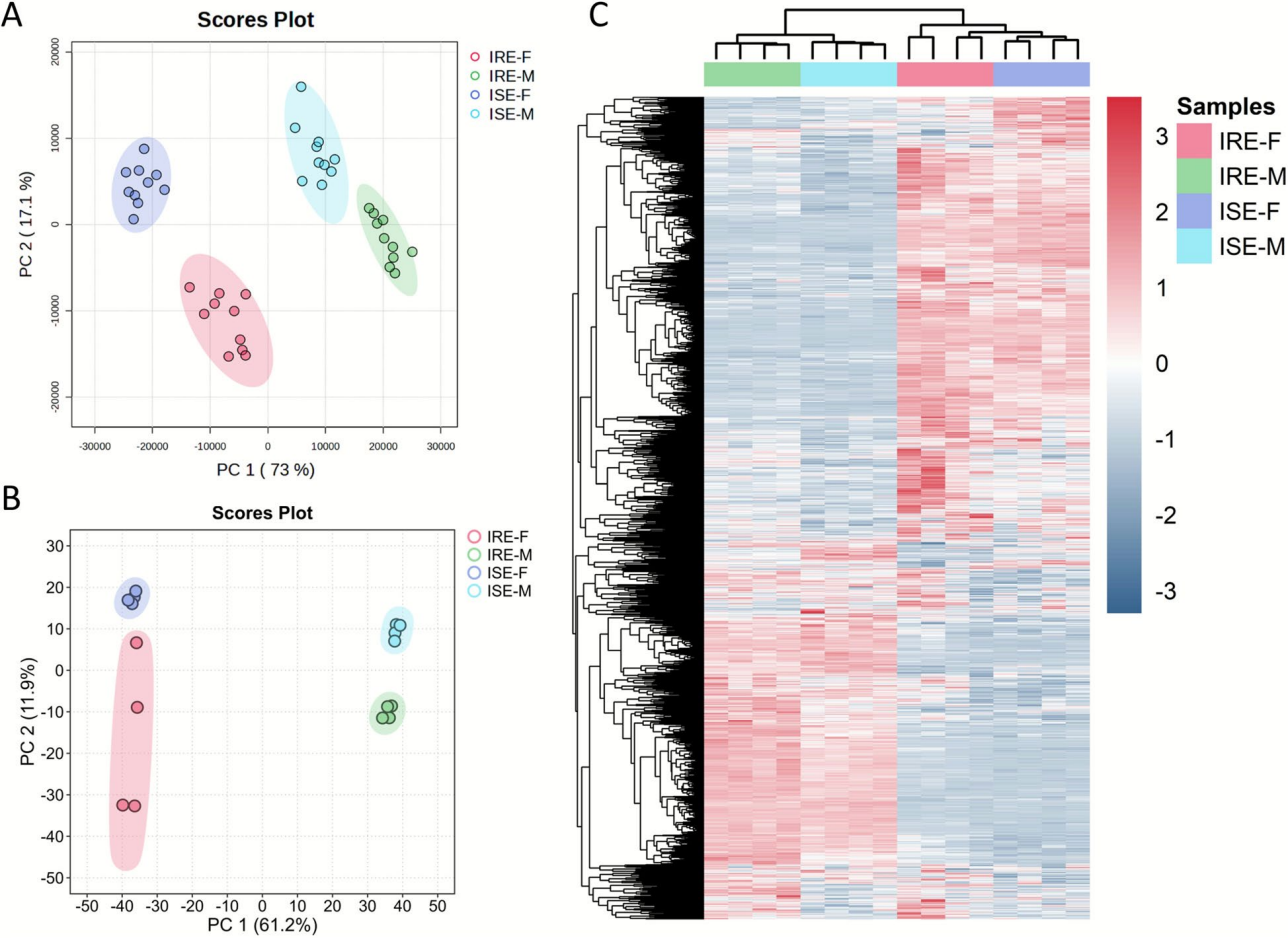
**Principal component analysis (PCA) score plots representing all experimental groups from **(**A**) the metabolome analysis (*n* = 10) and (**B**) the proteome analysis (*n* = 4). **C** Hierarchical clustering analysis displaying the expression profiles of all quantified protein abundances (clustering: Euclidean distance, complete linkage; *z*-score normalization). IRE-F, resistant females; IRE-M, resistant males; ISE-F, sensitive females; ISE-M, sensitive males.

To obtain an initial understanding of the effects of anthelmintic resistance and sex on metabolite levels, we first performed an untargeted analysis of binned spectral data using multivariate statistical approaches. A common principal component analysis (PCA) model comprising all four experimental groups revealed distinct separation according to both sex and resistance status (Figure 1A). Subsequently, two separate PLS-DA models were constructed to assess the impact of anthelmintic resistance independently in males and females. Robustness of both models was confirmed through leave-one-out cross-validation (LOOCV) and permutation testing. The respective score plots and comprehensive validation results are available in Additional file 2. Bins associated with metabolites responsible for distinguishing between males and females, as well as between resistant and sensitive groups, were identified on the basis of VIP scores greater than 1. A consistent trend emerged across both male and female models, characterized primarily by a reduction in the concentrations of several amino acids (valine, isoleucine, phenylalanine, tyrosine, and proline), along with an increased level of trehalose and dimethylamine in the resistant compared with the sensitive group.

To gain a deeper insight into the differences in metabolite levels between the individual groups, we performed a univariate analysis on all metabolites identified and quantified across all sample extracts. In the ^1^H NMR spectra of polar extracts from *H. contortus*, we analyzed a total of 79 signals or parts of multiplets, which were subsequently unambiguously assigned to 39 metabolites (Additional file 3). Identifications were further supported by the chemical shifts of selected carbon atoms, indirectly observed in the HSQC experiment. Minor signals that could not be reliably assigned were excluded from the analysis.

First, our assumption that significant differences exist between males and females was confirmed, emphasizing the necessity to evaluate the effect of resistance separately for males and females. Statistical testing (as described in the Experimental section) was performed following confirmation of data normality (Shapiro–Wilk test of normality). Qualitative differences in the metabolome were already apparent upon visual inspection of the spectra, most notably the absence of signals corresponding to myo-inositol, asparagine, and aspartate, and the presence of dimethylglycine, which were characteristic features of all male spectra.

The full results of the targeted univariate analysis are presented in Table 1, which lists all metabolites significantly affected by sex or resistance. Differences between males and females followed similar metabolic patterns across both the resistant and sensitive strains. Compared with males, females exhibited not only the presence of asparagine, aspartate, and myo-inositol but also significantly elevated levels of various amino acids (e.g., glutamine, isoleucine, leucine, valine, lysine, methionine, phenylalanine, proline, and tyrosine), tricarboxylic acid (TCA) cycle intermediates (fumarate and malate), phosphoenolpyruvate, isovalerate, and an unspecified UDP-saccharide. Conversely, both female groups showed decreased levels of lactate, propionate, *sn*-glycero-3-phosphocholine, *O*-acetylcholine, betaine, and dimethylamine.

**Table 1:** **Significantly changed metabolites**

Metabolite	Resistant versus sensitive				Females versus males			
	Females		Males		Sensitive		Resistant	
	∆%	*p*-Value	∆%	*p*-Value	∆%	*p*-Value	∆%	*p*-Value
Asparagine	17.0	***	n.e.		n.e.		n.e.	
Aspartate	53.1	****	n.e.		n.e.		n.e.	
Glutamine	72.5	****	12.2	ns	61.6	****	148.4	****
Glycine	2.7	ns	1.7	ns	16.2	*	17.3	**
Histidine	−22.3	*	8.8	ns	74.6	****	24.6	
Isoleucine	−38.2	****	−43.6	****	41.6	****	55.1	****
Leucine	−36.5	****	−40.0	****	67.9	****	77.6	****
Valine	−35.5	****	−36.9	****	70.9	****	74.8	****
Lysine	−22.3	****	−14.3	*	82.7	****	65.8	****
Methionine	−32.6	****	−32.6	****	42.7	****	42.7	****
Phenylalanine	−35.2	****	−41.5	****	83.1	****	102.6	****
Proline	−7.6	****	−9.2	*	122.0	****	125.8	****
Threonine	−0.4	ns	−27.8	****	−0.5	ns	37.3	****
Tryptophan	−22.2	**	−33.2	***	21.8	*	41.8	**
Tyrosine	−28.4	****	−40.7	****	50.9	****	82.3	****
Fumarate	−15.8	*	−16.4	ns	144.1	****	146.0	****
Succinate	−29.3	*	−41.2	**	20.5	ns	45.0	ns
Malate	−18.3	*	−25.2	*	44.9	***	58.2	***
Glucose	13.9	ns	0.8	ns	−23.7	**	−13.9	ns
Lactate	11.7	ns	6.0	ns	−26.5	**	−22.5	*
Phosphoenolpyruvate	14.8	*	42.6	**	106.2	****	66.1	****
Acetate	3.8	ns	−1.0	ns	4.4	ns	9.4	*
Formate	182.6	***	29.2	ns	−35.5	ns	41.0	ns
Propionate	6.0	ns	−20.9	ns	−53.6	****	−37.8	**
Isovalerate	5.8	ns	9.5	ns	134.6	****	126.8	****
*sn*-Glycerophosphocholine	7.8	**	10.2	****	−35.0	****	−36.4	****
*O*-acetylcholine	1.5	ns	−2.1	ns	−35.7	****	−33.4	****
*O*-phosphocholine	−0.6	ns	−28.6	****	−9.0	*	26.6	****
Betaine	13.8	***	−1.0	ns	−46.1	****	−38.0	****
Dimethylamine	209.5	****	178.2	****	−32.9	**	−25.4	****
NAD^+^	43.7	****	1.0	ns	−19.4	**	14.6	*
Trehalose	31.3	****	18.3	****	−8.7	*	1.4	ns
Myo-inositol	13.6	****	n.e.		n.e.		n.e.	
UDP-saccharide	−1.3	ns	−14.4	****	14.7	****	32.3	****

The results are expressed as the percentage change of normalized concentrations in resistant versus sensitive strains or females versus males (*n* = 10). The statistical significance was analyzed by two-way ANOVA, followed by Tukey’s post hoc test and Benjamini–Hochberg correction. * *p* < 0.05; ** *p* < 0.01; *** *p* < 0.001; **** *p* < 0.0001; ns, not significant; n.e., not evaluated

Comparing the metabolite levels in adults from the ISE and IRE strains, significant differences were identified. Most metabolic alterations associated with resistance were similar across sexes, although they arose from different baseline metabolite levels. In both females and males of the IRE strain, a marked decrease in the concentrations of most amino acids (including isoleucine, leucine, valine, lysine, methionine, phenylalanine, proline, tryptophan, and tyrosine) and TCA cycle intermediates (fumarate, succinate, and malate) was observed, accompanied by increased levels of trehalose, phosphoenolpyruvate, and dimethylamine. Nevertheless, the adults of the IRE strain also exhibited sex-specific metabolic responses compared with the sensitive group. These included a significant decrease in histidine levels in females, reduced concentrations of threonine and *O*-phosphocholine in males, and elevated levels of betaine and NAD^+^ in females.

### Proteome analysis

Following the findings of substantial sex- and resistance-related metabolic alterations in *H. contortus*, we further analyzed the differences in the proteomes of the two strains (ISE and IRE) using isobaric labeling with tandem mass tags (TMT) followed by high-resolution LC–MS/MS. A total of 3014 unique proteins were quantified, of which 2836 proteins were quantified with at least two valid values (Additional file 4). Similar to our metabolome results, principal component analysis and hierarchical clustering distinguished all four sample groups, with clear differences between sexes and smaller differences between strains (Figures 1B, C).

**Figure 2:**
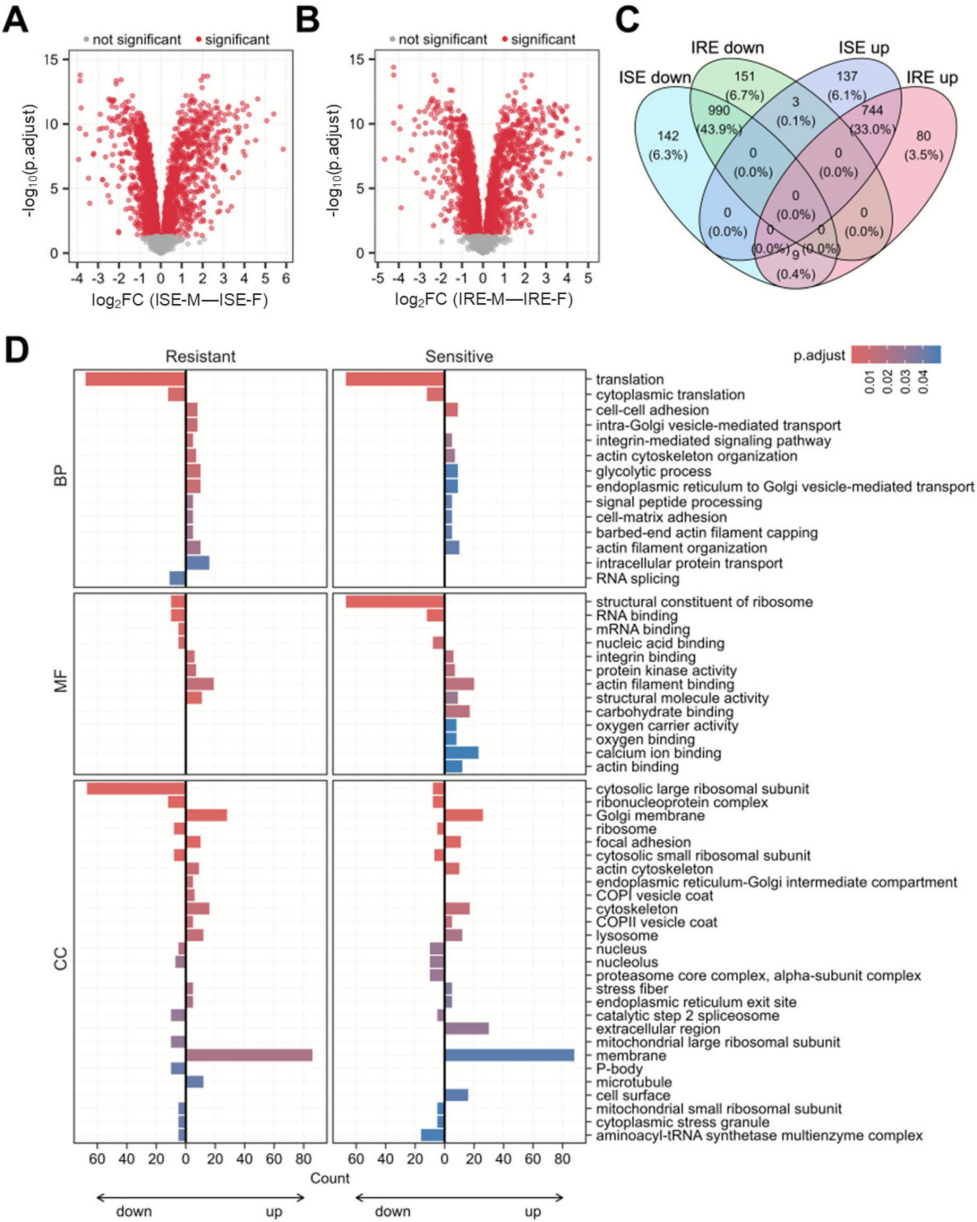
**Sex-related differences in the protein expression of sensitive** (**A**) and resistant (**B**) adults of *H. contortus*. Log_2_ fold changes (FC) show the difference in the protein expression in males compared with females of the corresponding strain. Significant proteins with the adjusted *p*-value < 0.05 are highlighted in red. **C** Venn diagram of differentially expressed proteins from (**A**) and (**B**). **D** Over-representation analysis (ORA) of Gene Ontology (GO) terms, including biological process (BP), molecular function (MF), and cellular compartment (CC), of all sex-related DEPs. Significantly up- and downregulated proteins were tested separately. The counts of term-specific enriched proteins on the right relative to 0 on the *x*-axis show upregulated pathways, while the counts of proteins to the left represent downregulated pathways. The expression in males was compared with the expression in females of the corresponding strain (log_2_ FC [males–females]). ORA was performed using the experiment-specific protein background and a Benjamini–Hochberg FDR of 5%. IRE, resistant adults; ISE, sensitive adults.

**Figure 3:**
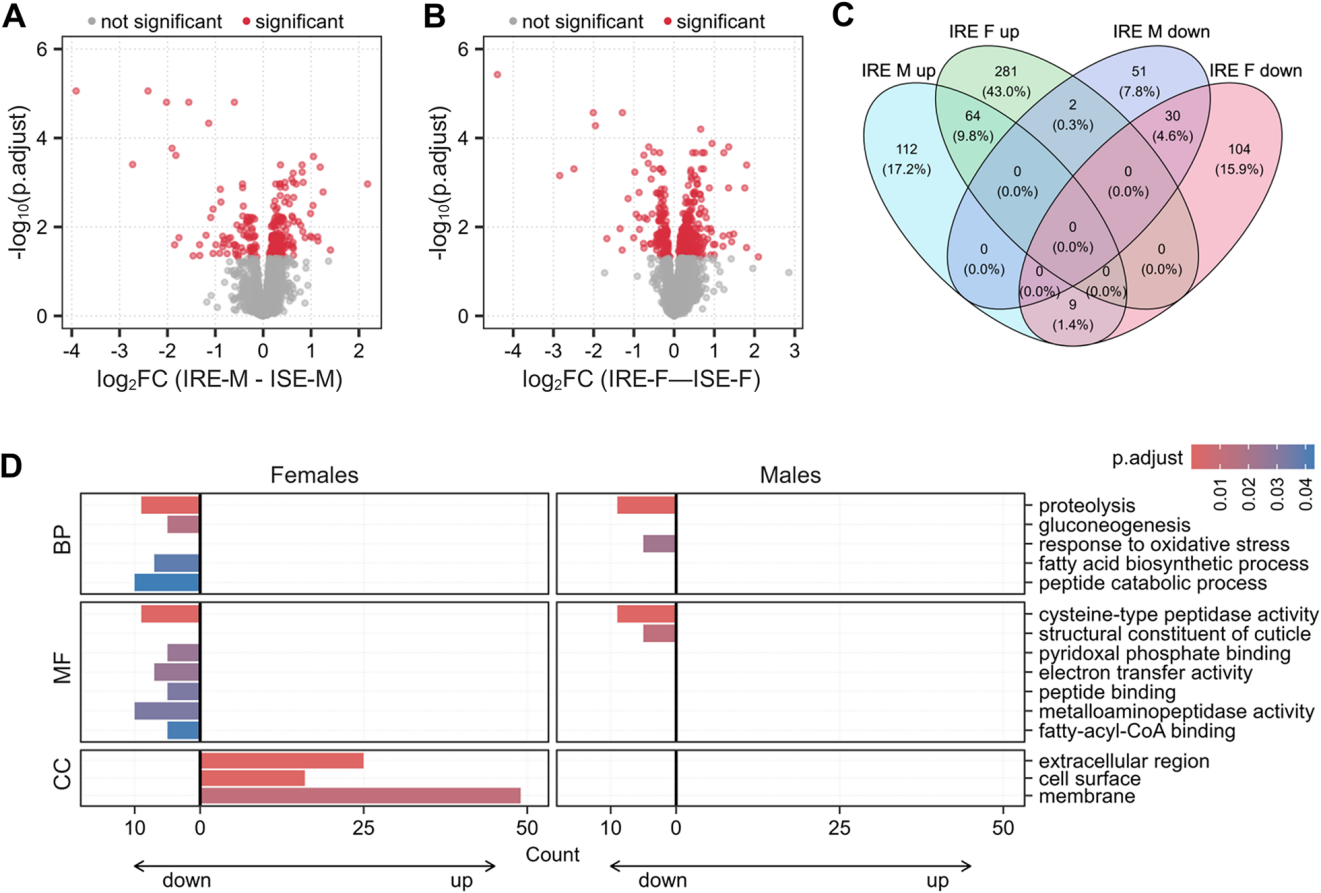
**Resistance-related differences in the protein expression in males** (**A**) and females (**B**) of *H. contortus*. Log_2_ fold changes show the difference in the protein expression in resistant adults compared with sensitive adults. Significant proteins with the adjusted *p*-value < 0.05 are highlighted in red. **C** Venn diagram of differentially expressed proteins from (**A**) and (**B**). **D** Over-representation analysis (ORA) of Gene Ontology (GO) terms, including biological process (BP), molecular function (MF), and cellular compartment (CC), of all resistance-related DEPs. Significantly up- and downregulated proteins were tested separately. The counts of term-specific enriched proteins on the right relative to 0 on the *x*-axis show upregulated pathways, while the counts of proteins to the left represent downregulated pathways. The expression in resistant adults was compared with sex-matched sensitive adults (log_2_ FC [resistant–sensitive]). ORA was performed using the experiment-specific protein background and a Benjamini–Hochberg FDR of 5%. IRE, resistant adults; ISE, sensitive adults.

Differential expression analysis confirmed substantial sex-associated differences in the global proteome profiles of *H. contortus* adults (Figures 2A, B), further underscoring the importance of carefully considering sex-specific characteristics of this parasitic nematode. The differences between sexes were consistent across both analyzed strains of *H. contortus*, with a large proportion (~77%) of jointly differentially expressed proteins (DEPs) (Figure 2C). However, global proteome changes related to anthelmintic resistance were rather moderate (Figures 3A, B), with the considerable sex-dependent adaptations apparent also in the context of resistance development. Only ~14% of all DEPs were up/downregulated simultaneously in both sexes (Figure 3C).

Owing to limited annotations of *H. contortus* proteins, all sex- and resistance-associated DEPs were used for the following functional over-representation analysis (ORA) of Gene Ontology (GO) terms. In addition, ORA was performed separately for significantly downregulated and upregulated proteins. In the context of the sex-associated differences, consistent enrichment patterns centered around translational regulation and cytoskeletal organization (Figure 2D). Specifically, in males of both strains, GO terms such as translation, ribosomes, RNA binding, ribonucleoprotein complex, aminoacyl-tRNA synthetase multienzyme complex, etc. were significantly downregulated. In contrast, actin cytoskeleton organization, actin filament organization, actin filament binding, and other GO terms related to cytoskeleton-associated processes, such as intracellular transport (e.g., intra-Golgi vesicle-mediated transport, endoplasmic reticulum to Golgi vesicle-mediated transport, intracellular protein transport, and COPI and COPII vesicle coats) and cell adhesion and signal transduction (e.g., cell–cell adhesion, integrin-mediated signaling pathway, cell–matrix adhesion, etc.) were significantly upregulated in males compared with females from both studied strains. Overall, these findings indicate that sex-specific proteomic differences in *H. contortus* adults are shaped by a coordinated downregulation of translational machinery and upregulation of cytoskeletal dynamics in males relative to females.

The resistance-associated adaptations differed between resistant females and resistant males; however, both sexes shared a common mechanism characterized by reduced proteolysis and cysteine-type peptidase activity (and also metalloaminopeptidase activity in the resistant females). Interestingly, in resistant females, downregulated proteins were strongly linked to several metabolic pathways, namely, fatty acid biosynthesis, pyridoxal phosphate binding, electron transfer activity, and fatty-acyl-CoA binding. Collectively, the results suggest that considerable metabolic shifts, reduced protein turnover, and more efficient substrate utilization might play an important role in the development of resistance, especially in females (Figure 3D).

On the basis of the metabolome analysis we searched a KEGG database for all pathways and enzymes that may be responsible for the differences in metabolite quantities observed. These pathways were crossed with the *Caenorhabditis elegans* genome, because of its better level of annotation, and the proteins predicted to be involved were then used to search for homologous proteins in *H. contortus* (Additional file 4). Using the identified set of protein IDs, we searched differentially expressed proteins, particularly those with connections to the metabolites found in significantly different levels between the ISE and IRE strains. These selected proteins (Table 2) are discussed further, together with transthyretin-like proteins, which represent an interesting protein family without connection to out metabolome results but with an increased expression of approximately 30 isoforms in IRE females in comparison with ISE females. All proteins can be found in Additional file 4.

**Table 2:** **Selected proteins with connections to the metabolites with different levels in *H. contortus*** **adults of the ISE and IRE strains.**

Accession	Short_description	Resistant versus sensitive females			Resistant versus sensitive males		
		Log_2_ FC	Adjusted *p*-value	Expression	Log_2_ FC	Adjusted *p*-value	Expression
A0A7I5E8B1	Alanine–glyoxylate aminotransferase	0.339	0.003	Up	0.171	0.097	ns
A0A6F7Q0I3	Cysteine synthase	0.230	0.016	Up	0.084	0.391	ns
A0A7I4XWI2	Glutathione reductase	0.166	0.016	Up	0.246	0.003	Up
A0A7I4Y2V0	Histidine ammonia-lyase	0.196	0.015	Up	0.134	0.078	Up
A0A7I4Z1Y2	Isocitrate dehydrogenase	0.150	0.017	Up	0.218	0.003	Up
A0A7I5E6Q5	Isocitrate dehydrogenase	0.206	0.011	Up	0.147	0.061	ns
A0A7I4XXX3	Isocitrate dehydrogenase	0.387	0.017	Up	0.261	0.110	ns
A0A7I4XSV1	Phosphoenolpyruvate carboxykinase (GTP)	−0.752	0.000	Down	−0.450	0.010	Down
A0A7I5E8L5	Phosphoenolpyruvate carboxykinase (GTP)	−0.103	0.047	Down	−0.087	0.118	ns
A0A7I4YLE1	Proline dehydrogenase	0.295	0.010	Up	0.241	0.033	Up
A0A7I4YQ11	Pyruvate kinase	−0.347	0.006	Down	−0.258	0.032	Down
A0A7I5E5I0	Pyruvate kinase	0.204	0.039	Up	−0.063	0.608	ns
A0A7I4XTX9	Pyruvate kinase	0.308	0.041	Up	0.215	0.181	ns
A0A7I5E5J1	Pyruvate kinase	0.432	0.032	Up	0.178	0.421	ns

## Discussion

Despite decades of research into anthelmintic resistance, most current knowledge centers around a set of well-characterized target-site mechanisms, such as mutations in β-tubulin, ligand-gated chloride channels, or nicotinic acetylcholine receptors [13]. In addition, some nontarget site mechanisms, e.g., increased expression of efflux transporters P-glycoproteins, cytochromes P450, and UDP-glycosyltransferases, have been also revealed [24, 41]. However, the newest transcriptomic and genomic data suggest that additional yet unidentified molecular pathways may be contributing to treatment failure. Uncovering these novel mechanisms is critical for several reasons. First, reliance on a narrow set of resistance markers may lead to underdiagnosis of resistance in populations where alternative pathways dominate. Second, emerging evidence indicates that resistance may involve complex polygenic traits, epigenetic regulation, or interactions with the parasite microbiome, which are not captured by current diagnostic tools. Third, novel resistance mechanisms may compromise the efficacy of future drug candidates if they share structural or functional similarities with existing compounds [42]. Recent advances in high-throughput sequencing, transcriptomics, proteomics, and metabolomics offer unprecedented opportunities to explore these unknown pathways [23, 43].

Metabolomics can identify biochemical adaptations, such as altered energy metabolism, enhanced detoxification, or lipid remodeling, that support parasite survival under drug pressure [44]. These metabolic shifts may serve as novel biomarkers or therapeutic targets. Moreover, metabolomic data can clarify the functional impact of resistance mutations and reveal compensatory pathways, especially in polygenic or noncanonical resistance contexts [42, 45]. To date, the only study focusing on the comparative metabolomic analysis of drug-resistant and drug-sensitive strains of *H. contortus* was published by Tuersong et al. in 2023 [26]. Using LC–MS, the authors analyzed mixed-sex samples from ivermectin-resistant and susceptible strains and concluded that ivermectin resistance is closely associated with amino acid metabolism.

In our study, comparative metabolomic profiling of adult *Haemonchus contortus* from the ISE and IRE strains revealed notable resistance-associated differences in the abundance of specific metabolites. While the most of resistance-related alterations were consistent across sexes, several changes were sex-specific (e.g., in multiple amino acids, formate, phosphocholine, glutamine, betaine, myo-inositol, and NAD^+^). These findings underscore the importance of analyzing females and males separately, despite the increased complexity and cost associated with such experiments. Metabolome results show that the resistant adults exhibit decreased levels of many amino acids and TCA cycle intermediates, but higher levels of phosphoenolpyruvate, formate, glutamine, and dimethylamine. *H. contortus* adults gain amino acids mainly from the host’s blood and use them for protein synthesis, lipid synthesis, synthesis of saccharides (via TCA cycle intermediates and phosphoenolpyruvate), and/or as source of energy. Formate is probably the waste product of carbon metabolism, while glutamine and dimethylamine aid in ammonium detoxification [46]. Our results might indicate that the resistant adults utilize amino acids from the host to obtain energy, lipids, and saccharides faster. In agreement with this hypothesis, higher amounts of glycerophosphocholine, and an interesting saccharide trehalose, were found in resistant adults in comparison with the sensitive ones.

Glycerophosphocholine (GPC) is an important product from phospholipids and the source of the neurotransmitter acetylcholine. In *Caenorhabditis elegans*, GPC has been shown to extend lifespan, improve exercise capacity during aging, enhance stress resistance, and suppress the accumulation of reactive oxygen species [47]. In the entomopathogenic nematode *Steinernema kraussei*, GPC plays an important role in stress resistance by activating the expression of the *daf-16* gene within the insulin/IGF-1 signalling (IIS) pathway [48]. DAF-16, one of the central transcription factors in *C. elegans*, translocates from the cytoplasm to the nucleus upon activation, where it regulates diverse cellular processes including metabolism, autophagy, DNA damage repair, apoptosis, and oxidative stress resistance [49]. Studies in long-lived *C. elegans* mutants demonstrated that the lifespan-extending effect of phosphatidylcholine supplementation overlaps with reduced IIS activity and is dependent on DAF-16 [50]. Similarly, myo-inositol, the major physiological form of the sugar alcohol inositol, was significantly increased in resistant females in our study. Yang et al. reported that myo-inositol extends lifespan and improves health indices in *C. elegans* by inhibiting PI3K activity, thereby attenuating IIS signalling and promoting subsequent DAF-16 activation [51]. NAD^+^, also markedly elevated in resistant females, is a key cofactor in metabolic homeostasis and serves as a rate-limiting substrate for sirtuin deacylases. Reduced NAD^+^ levels shorten *C. elegans* lifespan, whereas genetic or pharmacological restoration of NAD^+^ prevents age-related metabolic decline and promotes longevity [52]. In summary, our findings suggest that increased levels of GPC, together with sex-specific elevations of myo-inositol and NAD^+^, collectively promote longevity and health span through the activation of DAF-16, albeit via distinct molecular mechanisms.

Trehalose, a disaccharide of glucose found in diverse organisms, is suggested to act as a stress protectant against heat, cold, desiccation, anoxia, and oxidation. Treatment of *C. elegans* with trehalose extended the mean life span without any side effects [53]. Moreover, upon exposure to environmental contaminants, *C. elegans* showcased a significant trehalose increase, highlighting an adaptive response to environmental stress by augmenting trehalose synthesis [54]. DAF-16 has been demonstrated to promote starvation resistance by reprogramming carbon metabolism to favor trehalose production, thereby supporting survival through both energy provision and stress protection [55]. Notably, our results demonstrate for the first time that elevated trehalose levels may protect *H. contortus* against anthelmintic-induced stress, potentially contributing to the development of drug resistance in parasitic nematodes.

In the second part of our study, the proteome of *H. contortus* adults was analyzed with a particular focus on the resistance-related differences. Comparing the proteomes of drug-sensitive and drug-resistant nematodes allowed us to detect the differential expression of proteins involved in adaptive responses that contribute to resistance, offering insights into regulatory mechanisms of resistance [56, 57]. In our study, we focused on the enzymes that could take part in the synthesis, utilization, and/or degradation of those metabolites, with changed levels in the adults of resistant versus sensitive strains. Unfortunately, only a few enzymes with a clear connection to these metabolites were found owing to limited annotation and/or low abundance. However, some interesting crosslinks between the metabolome and proteome results were detected. For example, alanine–glyoxylate aminotransferase (AGT), catalyzing the transamination of alanine and glyoxylate to pyruvate and glycine, was upregulated in resistant females. In nematodes such as *C. elegans*, AGT is a mitochondrial and highly active enzyme, supporting the glyoxylate cycle. This enables nematodes to efficiently convert stored lipids into glucose, especially under nutrient-limited or stressful conditions [58]. Upregulation of AGT in resistant strain of *H. contortus* represents an advantage for the parasite, and it can be associated with the increased amounts of trehalose (formed from two glucoses) detected in the IRE strain.

In addition, three isoforms of pyruvate kinase (PK), which catalyzes the ATP-generated transformation of phosphoenolpyruvate to pyruvate, were upregulated in IRE females, and two isoforms of phoshoenolpyruvate carboxykinase (PEPCK), catalyzing the formation of oxalacetate from phoshoenolpyruvate, were downregulated in resistant adults. A higher amount of phosphoenolpyruvate could mean a higher gain of ATP under partially anaerobic conditions in the host’s abomasum in the IRE strain in comparison with the ISE strain. Similarly, in dauer larvae, a nonfeeding and developmentally arrested stage of the free-living nematode *C. elegans*, PK and other enzymes of anaerobic metabolism were upregulated, which indicated the switch of dauer larvae to low oxygen consumption [59].

The faster utilization of amino acids in IRE adults assumed from metabolomic data corresponds to the increased expression of proline dehydrogenase and histidine ammonia-lyase, the first enzymes in catabolism of these amino acids. In resistant adults, a higher expression of NAD^+^-dependent isocitrate dehydrogenases, which catalyze the oxidative decarboxylation of isocitrate to 2-oxoglutarate, was also detected. These enzymes represent the allosterically regulated rate-limiting step of the TCA cycle. However, other TCA enzymes and intermediates were rather downregulated in the IRE strain, which complicated the interpretation of these findings.

Other interesting enzymes with upregulated expression in the IRE strain are glutathione reductase (in adults of both sexes) and cysteine synthase (in females), enzymes involved in glutathione synthesis and metabolism. Glutathione (γ-glutamylcysteinglycin, GSH) plays an important role in the maintenance of the intracellular thiol redox state and in detoxification processes. The intracellular GSH level depends on glutathione reductase expression/activity as well as on GSH synthesis [60, 61]. Cysteine, a limiting amino acid for GSH synthesis, can be synthetized from *N*-acetylserine and sulfide by cysteine synthase but only in bacteria, plants, and nematodes (not in mammals). Interestingly, the genome of *C. elegans* contains three expressed genes of cysteine synthase which play different roles in resistance to hypoxia, hydrogen sulfide, and cyanide [62]. *C. elegans* resistance to hydrogen cyanide is mediated by cysteine synthase, which likely functions in metabolic pathways that inactivate cyanide [63]. Cysteine synthase activity was also confirmed in the homogenate of *H. contortus*. Upregulation of cysteine synthase expression in adults and its downregulation in the L3 larval stage suggests that the de novo pathway contributes to the cysteine requirement of mature *H. contortus* [64]. The observed upregulation of cysteine synthase and glutathione reductase in adults of the IRE strain may indicate their improved balancing under chemical and oxidative stress.

Besides the enzymes involved in endogenous metabolic pathways, some proteins localized probably on the parasite's surface also exhibited higher expression in females from the IRE strain in comparison with females from the ISE strain. The transthyretin-like family of proteins seem to be the most interesting, as approximately 30 of these related proteins were expressed at higher levels in resistant females. Transthyretin-like proteins, a large nematode-specific family, can suppress host immune responses, particularly by inhibiting reactive oxygen species. Their main functions include modulating host immune responses, supporting nematode development, and facilitating successful parasitism. These proteins are highly conserved across nematode species and are abundant in excretory/secretory products, making them promising targets for nematicides [65–67]. However, several proteolytic enzymes were downregulated in the females of the IRE strain in comparison with the ISE strain, which might indicate lower utilization of the host’s proteins. But the reason remains unclear.

In conclusion, our integrated metabolomic and proteomic comparison of adults of *H. contortus* from the ISE and IRE strains revealed many significant resistance-related differences, indicating substantial changes in several biochemical pathways and provides the first evidence of sex-specific mechanisms underlying drug resistance in this parasite. These findings reinforce the prevailing view that the emergence of anthelmintic resistance is a multifactorial and highly complex process, the full extent of which is yet to be elucidated. Among metabolites with enhanced levels in the IRE strain, trehalose and GPC, and their associated metabolic pathways, warrant particular attention, as they likely play a pivotal role in nematode adaptation to drug-induced stress conditions. In parallel, several nematode-specific enzymes upregulated in the IRE strain, such as cysteine synthases and transthyretin-like proteins, highlight potential molecular targets for the development of novel anthelmintics. Nonetheless, these findings call for comprehensive follow-up studies that integrate metabolomic and proteomic datasets and include detailed functional validation to fully elucidate the mechanisms involved.

## Supplementary Information

**Additional file 1: TMT experiment quality check.** **A** Normalization factors derived from summed raw peptide intensities. Only the intensities of TMT labeled peptides assigned uniquely to *H. contortus* were summed. **B** Correlation plot of normalized and log_2_ transformed protein intensities. IRE-F – resistant females; IRE-M – resistant males; ISE-F – sensitive females; ISE-M – sensitive males.

**Additional file 2: PLS-DA score plots of the resistant vs. sensitive strains separately for males (A) and females (B).** The leave-one-out cross-validation results for 2 principal components: accuracy = 1.00, R2 = 0.95, Q2 = 0.88 for both male and female models. IRE strain is marked in red, ISE in green.

**Additional file 3. A representative 1H NMR spectrum of polar extracts from** ***H. contortus*** **with the metabolite assignment.**

**Additional file 4: TMT experimental design (S1).** Complete list of proteins identified and quantified in *H. contortus* adults (S2). Differential expression analyses of their levels (S3) between resistant and susceptible females (S3a), resistant and susceptible males S3b), sensitive males and females (S3c), and resistant males and females (S3d). KEGG pathway analysis of detected metabolites against *C. elegans* genome (S4a). Homologous relationships for* H. contortus* proteins identified using BLAST bidirectional best hits (S4b).

## Data Availability

The NMR metabolomics data have been deposited to the ASEP repository [68]. The mass spectrometry proteomics data have been deposited to the ProteomeXchange Consortium [69] via the PRIDE partner repository [70] with the dataset identifier PXD067321. The experimental metadata was generated using lesSDRF [71].
