# Supplementary figures and images for: Metabolomic and proteomic differences in susceptible and benzimidazole-resistant adult females and males of Haemonchus contortus
Source: Vet Res. 2025 Dec 24;57:17. doi: 10.1186/s13567-025-01698-3 (PMC12849590; doi:10.1186/s13567-025-01698-3)

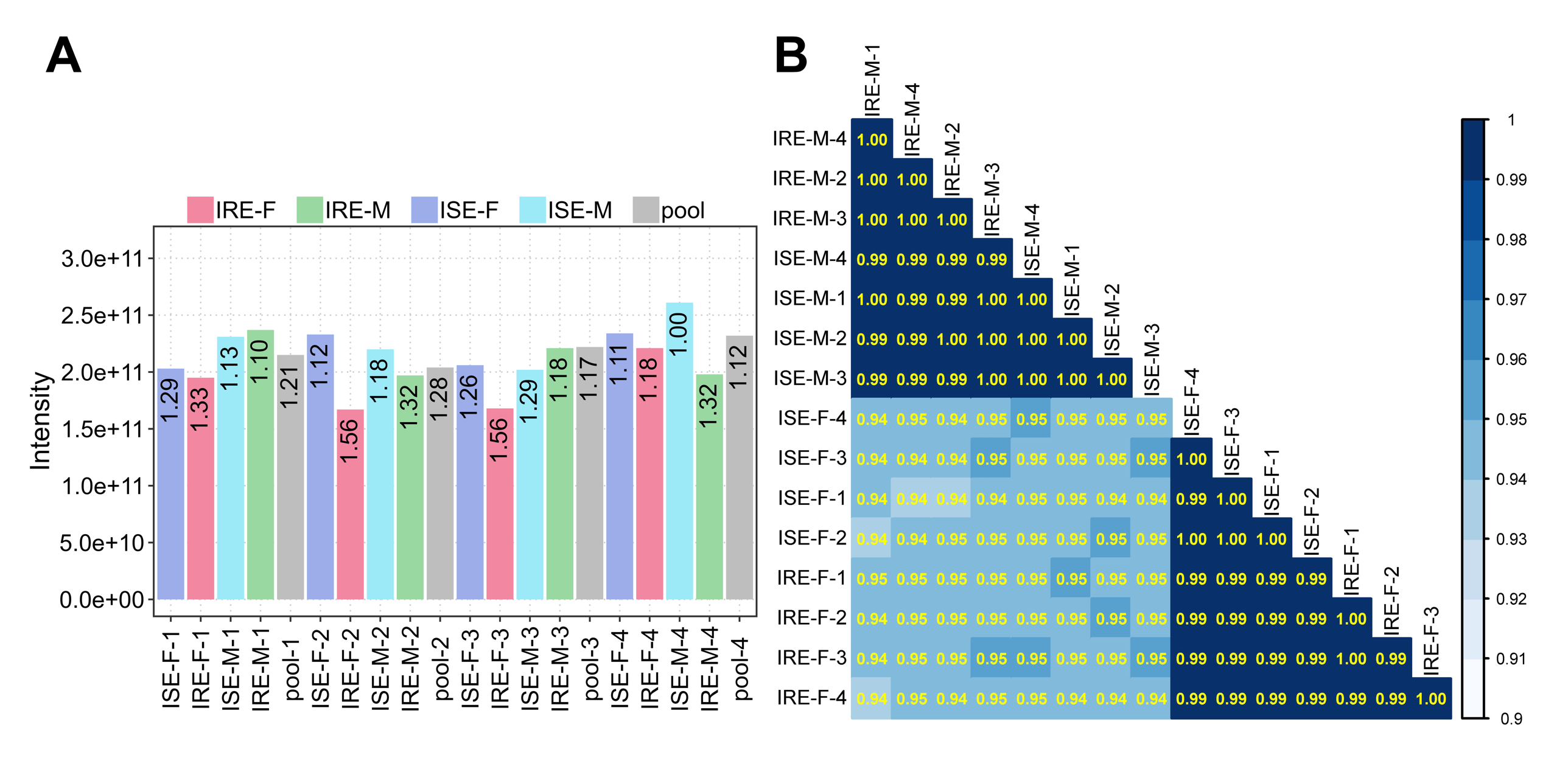

Supplement: Supplementary file 1 — Additional file 1: TMT experiment quality check. A Normalization factors derived from summed raw peptide intensities. Only the intensities of TMT labeled peptides assigned uniquely to H. contortus were summed. B Correlation plot of normalized and log2 transformed protein intensities. IRE-F – resistant females; IRE-M – resistant males; ISE-F – sensitive females; ISE-M – sensitive males. [file 13567_2025_1698_MOESM1_ESM.tif]

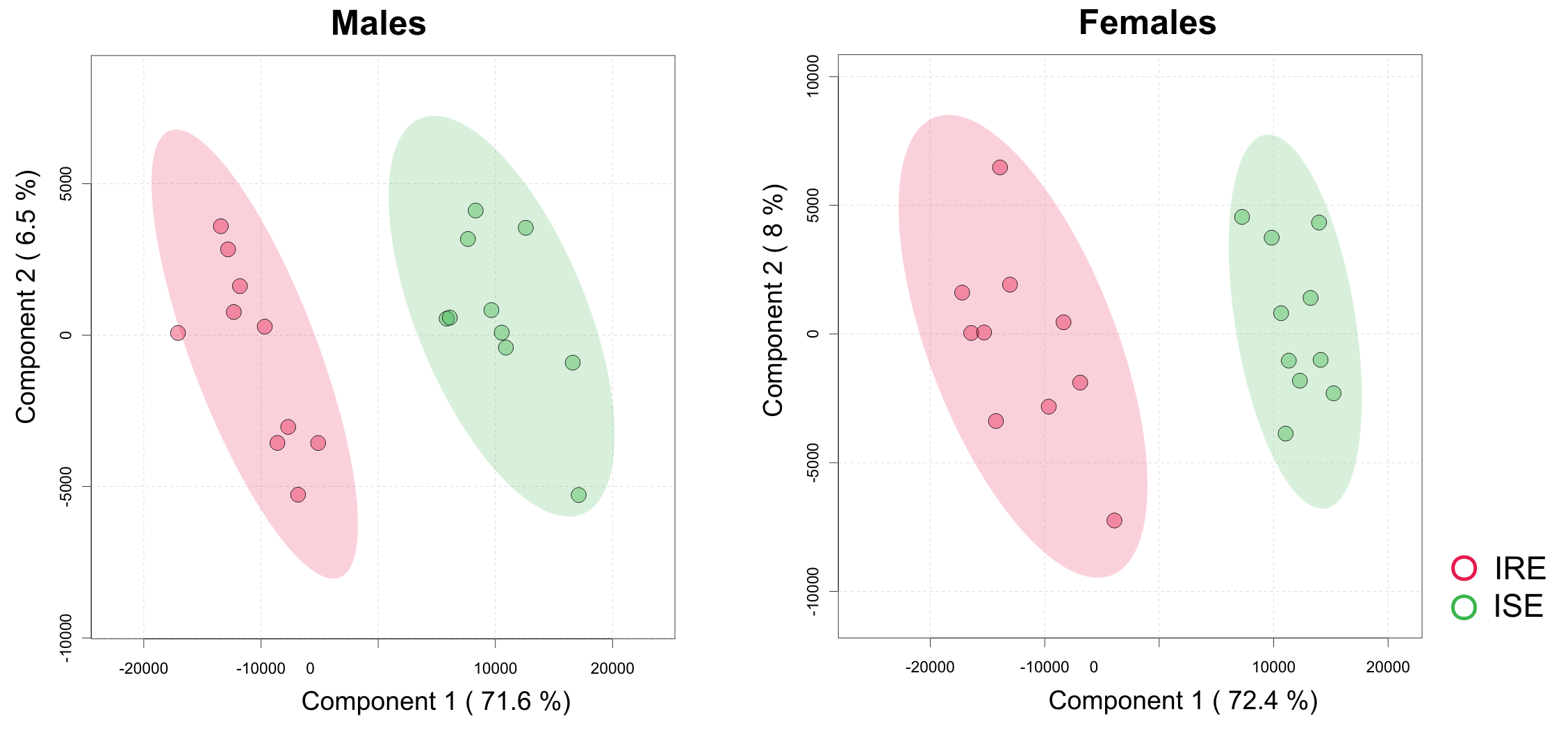

Supplement: Supplementary file 2 — Additional file 2: PLS-DA score plots of the resistant vs. sensitive strains separately for males (A) and females (B). The leave-one-out cross-validation results for 2 principal components: accuracy = 1.00, R2 = 0.95, Q2 = 0.88 for both male and female models. IRE strain is marked in red, ISE in green. [file 13567_2025_1698_MOESM2_ESM.tif]

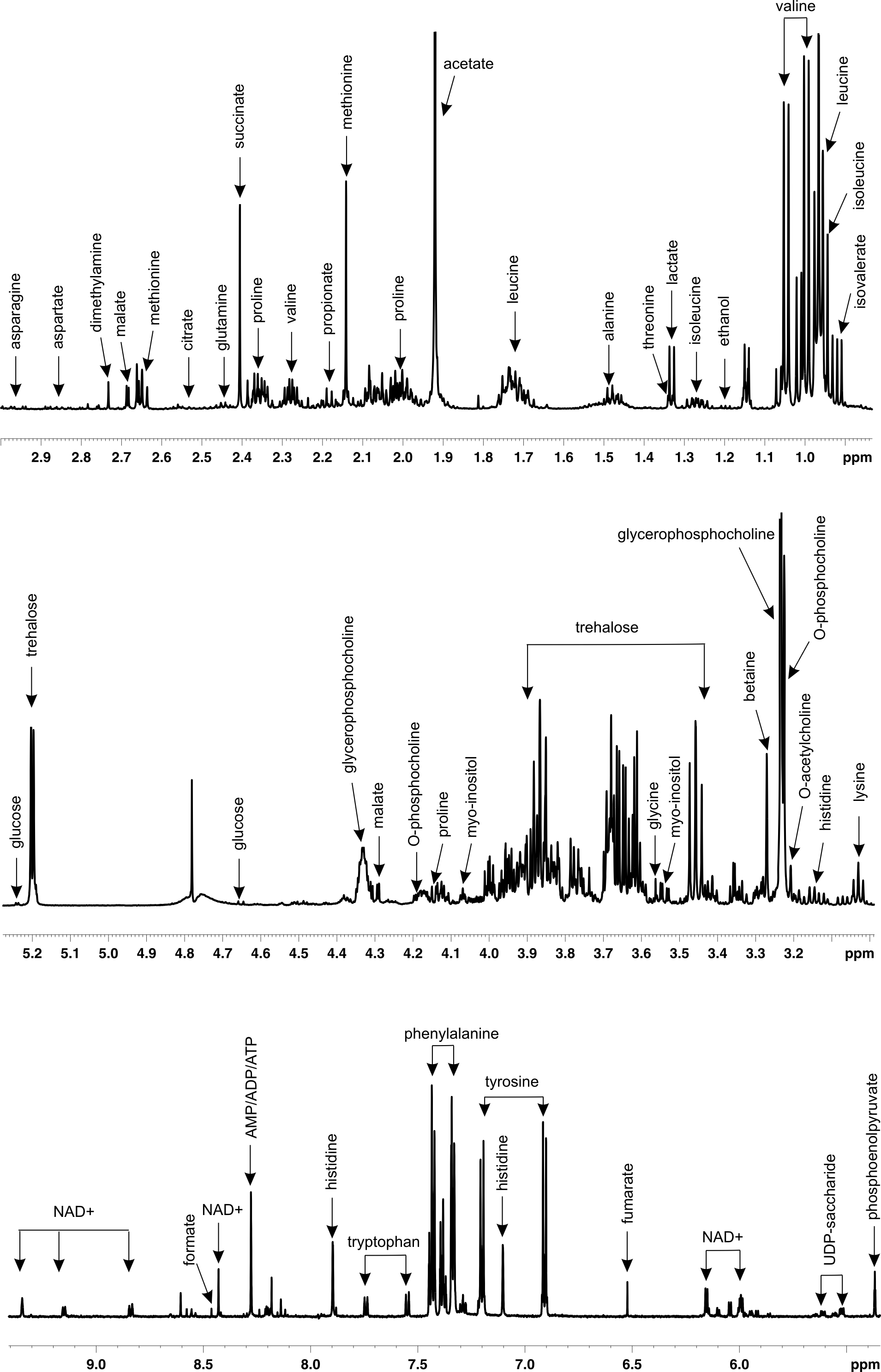

Supplement: Supplementary file 3 — Additional file 3. A representative 1H NMR spectrum of polar extracts from H. contortus with the metabolite assignment. [file 13567_2025_1698_MOESM3_ESM.tif]
